# Comparison of Contralateral Breast Background Parenchymal Enhancement on MRI Before and After Neoadjuvant Chemotherapy According to Molecular Subtypes in Unilateral Breast Cancer

**DOI:** 10.3390/diagnostics15222826

**Published:** 2025-11-07

**Authors:** Mi Young Kim, Nami Choi, Surin Park, Jeemin Seo, Su Yeon Ahn, Yoon Joo Shin

**Affiliations:** Department of Radiology, Konkuk University Medical Center, Konkuk University School of Medicine, Seoul 05030, Republic of Korea; 20120169@kuh.ac.kr (M.Y.K.); 20200185@kuh.ac.kr (S.P.); 20250124@kuh.ac.kr (J.S.); 20190199@kuh.ac.kr (Y.J.S.)

**Keywords:** breast, MRI, neoadjuvant chemotherapy

## Abstract

**Background/Objectives:** To evaluate changes in background parenchymal enhancement (BPE) in the contralateral breast on MRI before and after neoadjuvant chemotherapy (NAC), stratified by molecular subtype in patients with unilateral breast cancer. **Methods:** This study retrospectively analyzed 116 individuals diagnosed with unilateral breast cancer by biopsy, all of whom underwent breast MRI examinations before and after neoadjuvant chemotherapy. Contralateral breast BPE was graded into four levels (BPEC: 1 = minimal, 2 = mild, 3 = moderate, 4 = marked) by two readers in consensus. Histopathological features and BPE reduction were compared according to molecular subtype. **Results:** BPE showed a reduction across all molecular subtypes after NAC. In ER-positive cancers, BPEC shifted from 26/16/28/30% to 68/28/4/0%; in HER2-positive cancers, from 37.8/26.7/22.2/13.3% to 73.3/20.0/6.7/0%; and in triple-negative breast cancers, from 47.6/14.3/23.8/14.3% to 76.2/14.3/9.5/0%. Compared to the ER-positive cancer, the reduction in BPE over time was significantly greater in the HER2-positive cancer group (Estimate = 0.48, *p* = 0.0168) and TNBC (Estimate = 0.55, *p* = 0.0321), suggesting that the extent of BPE decrease varied by subtype. **Conclusions:** The extent of BPE reduction on breast MRI following NAC varies significantly across different molecular subtypes of breast cancer.

## 1. Introduction

Background parenchymal enhancement (BPE) represents the degree of contrast uptake in the fibroglandular tissue on breast MRI and is graded as minimal, mild, moderate, or marked according to the BI-RADS^®^ lexicon [1]. Clinically BPE is affected by factors including patient age and hormonal status [2]. Multiple retrospective case–control and cohort analyses have assessed the correlation between increased qualitative BPE and subsequent breast cancer diagnosis. Several studies indicated that higher BPE is strong predictor of breast cancer in high-risk women [3,4,5], and a large retrospective analysis from the Breast Cancer Surveillance Consortium, including 4247 women, found that those with mild or higher BPE had an elevated risk of subsequent breast cancer [6]. In a multicenter case–control study involving 835 women with breast cancer and 963 controls undergoing diagnostic, screening, or surveillance breast MRI, premenopausal women with moderate or marked BPE had a higher likelihood of breast cancer compared to those with minimal or mild BPE [7].

Breast cancer can be classified into three immunohistochemistry (IHC)-based molecular subtypes [8]: estrogen receptor (ER) positive (and human epidermal growth factor receptor 2 [HER2] negative, independent of progesterone receptor [PR] status), HER2 positive (independent of ER and PR status), and “triple negative” (ER negative, PR negative, and HER2 negative). The pattern of BPE on breast MRI may be associated with distinct immunohistochemical characteristics and receptor status across breast cancer subtypes. Dilorenzo et al. reported that mild BPE is often observed in patients with Luminal B HER2-negative breast cancer, whereas marked BPE tends to be associated with triple-negative breast cancer (TNBC) [9]. Velden et al. reported that parenchymal enhancement in the contralateral breast of patients with invasive unilateral breast cancer is significantly associated with long-term outcome, particularly in patients with ER-positive, HER2-negative breast cancer [10].

Neoadjuvant chemotherapy (NAC) is increasingly employed in breast cancer management, with MRI being the preferred imaging technique for assessing treatment response [11,12]. Numerous investigations have evaluated the relationship between BPE on breast MRI and tumor response to NAC. Collectively, these studies have demonstrated that the magnitude of BPE reduction—whether measured quantitatively or qualitatively—is associated with the pathological response to NAC in patients with breast cancer [13,14,15,16,17,18]. You et al., especially, reported that a greater early reduction in BPE during NAC was associated with a higher likelihood of achieving pathologic complete remission (pCR), particularly among patients with hormone receptor-negative tumors [18].

However, evidence remains limited regarding whether these changes differ according to molecular subtype. Given the biological heterogeneity of breast cancer and the distinct treatment responses observed across subtypes, understanding subtype-specific patterns of BPE reduction during NAC may provide additional insights into tumor biology and therapeutic response. In this context, we hypothesized that molecular subtype-specific differences in BPE reduction would be observed, thereby highlighting BPE as a potential imaging biomarker for predicting therapeutic response in breast cancer. To minimize the potential influence of ipsilateral tumorigenesis on BPE assessment, we analyzed the contralateral breast in patients with unilateral breast cancer. Therefore, the purpose of this study was to assess alterations in contralateral breast BPE on MRI before and after NAC, stratified by molecular subtype, in patients with unilateral breast cancer.

## 2. Materials and Methods

### 2.1. Patient Collection

**Figure 1:**
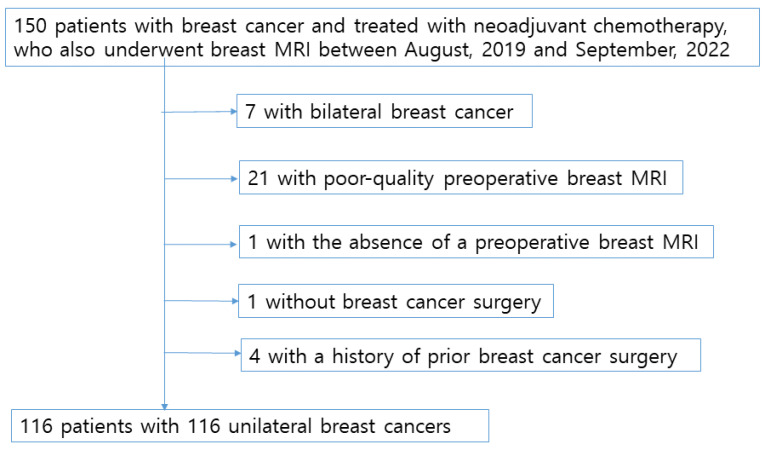
Schematic representation of the patient enrollment process.

The study was approved by the Institutional Review Board (IRB) of Konkuk University Medical Center (KUH 2025-06-012). A schematic diagram was used to illustrate the patient enrollment process (Figure 1). A total of 150 breast cancer patients who received NAC and underwent breast MRI at our institution between August 2019 and September 2022 were identified from the electronic medical records of Konkuk University Medical Center, Seoul, Korea. Of the initial 150 patients, 7 with bilateral breast cancer were excluded, and 21 were excluded due to poor-quality preoperative breast MRI images obtained from outside institutions. One patient was excluded because no surgery was ultimately performed, and another was excluded due to the absence of a preoperative breast MRI. Furthermore, four patients with a history of prior breast cancer surgery were excluded from this study, as BPE on MRI could be affected by adjuvant hormonal therapy administered after surgery. Consequently, 116 patients were included in the final analysis. Thus, our study cohort consisted of 116 patients, each with a unilateral breast cancer. The average age of the patients at breast cancer diagnosis was 49.4 years, with a range of 27–69 years and a median age of 50 years. The pre- or postmenopausal status of the patients was noted, as were the tumor characteristics, such as the cancer type (invasive ductal, invasive lobular or other carcinomas), tumor size, nodal status, and hormone receptor status (ER, PR, and HER2). The patients’ ER, PR, and HER2 statuses were determined by immunohistochemical analysis. For the immunohistochemical analysis, formalin-fixed, paraffin-embedded tissue sections were immunohistochemically stained. The Allred score was used to determine the ER and PR statuses. The results were classified as positive when the total score, expressed as the sum of the proportion and immuno-intensity scores, was 3 or more. With regard to the HER2 evaluation, tumors with a 3+ score were classified as HER2-positive and tumors with a 0 or 1+ score were classified as negative. In tumors with a 2+ score, gene amplification using SISH (silver in situ hybridization) was used to determine the HER2 status. The HER2 expression was considered positive if the ratio of the HER-2 gene copies to the chromosome 17 signal was >2.

### 2.2. Breast Magnetic Resonance Imaging (MRI) Technique

Breast MRI examinations were performed using two different 3.0T scanners: Discovery MR750 (GE Healthcare, Milwaukee, WI, USA) with an 8-channel breast coil, and MAGNETOM Vida (Siemens Healthineers, Erlangen, Germany) with an 18-channel breast coil. The imaging protocol included axial diffusion-weighted imaging (DWI), sagittal T2-weighted imaging with fat suppression (T2 FS), and sagittal dynamic contrast-enhanced T1-weighted three-dimensional fat-suppressed imaging (T1 3D FS) obtained before and at five time points after contrast administration.

For the GE system, the acquisition parameters were as follows:

DWI: TR/TE = 7000/50.9 ms, slice thickness = 5 mm, no interslice gap, field of view (FOV) = 340 mm, matrix = 128 × 128.

Sagittal T2 FS: TR/TE = 4346/102.5 ms, slice thickness = 2 mm, no gap, FOV = 200 mm, matrix = 256 × 160.

Sagittal T1 3D FS dynamic: TR/TE = 4.4/1.8 ms, flip angle = 10°, slice thickness = 1 mm, FOV = 200 mm, matrix = 320 × 230.

For the Siemens system, the parameters were:

DWI: TR/TE = 4000/53.2 ms, slice thickness = 5 mm, no gap, FOV = 340 mm, matrix = 160 × 96.

Sagittal T2 FS: TR/TE = 3910/72 ms, slice thickness = 2 mm, no gap, FOV = 200 mm, matrix = 288 × 181.

Sagittal T1 3D FS dynamic: TR/TE = 5.9/2.5 ms, flip angle = 10°, slice thickness = 1 mm, FOV = 200 mm, matrix = 320 × 224.

A gadolinium-based contrast medium (Dotarem; Guerbet, Villepinte, France) was injected intravenously at a dose of 0.1 mmol/kg body weight, and the first post-contrast phase was acquired 40 s after injection, followed by subsequent 4 phases obtained at 90 s intervals. All MRI scans were collected and sent to a dedicated Breast MR workstation. Reconstruction images are obtained with Maximal intensity projection (MIP) image with subtraction using dynamic contrast enhanced T1WI in the early phase (90 s after contrast injection).

### 2.3. Data Analysis

All imaging studies from the 116 patients included in this investigation were evaluated by two attending radiologists, MY Kim and N Choi, who possessed thirteen and eighteen years of experience, respectively, in the interpretation of breast MRI. Both readers were aware that the patients had undergone NAC for breast cancer and performed a consensus review to assess alterations in BPE following treatment. Details on the interobserver agreement between the two readers for BPE grading are presented in the Supplementary Materials. MIP images with subtraction were employed for this analysis. BPE was classified into four ordinal categories (BPEC 1–4) representing minimal, mild, moderate, and marked enhancement, respectively.

NAC was administered according to institutional protocols and tailored to the molecular subtype of each tumor. The regimens included the following: AC→T (doxorubicin and cyclophosphamide followed by a taxane), ACHP (doxorubicin, cyclophosphamide, trastuzumab, and pertuzumab), ACP (doxorubicin, cyclophosphamide, and a taxane), TCHP (docetaxel, carboplatin, trastuzumab, and pertuzumab), and THP (docetaxel, trastuzumab, and pertuzumab). Selection of the specific regimen was determined by the treating oncologists based on molecular subtype, patient characteristics, and current clinical guidelines.

### 2.4. Statistical Analysis

All data are reported as counts and corresponding percentages. Covariates including age and menopausal status (premenopausal vs. post-menopausal) were predefined based on existing literature and clinical relevance. These variables were included in the linear mixed model (LMM) as fixed effects to adjust for potential confounding. Given the repeated-measures design with BPE values assessed before and after NAC, a linear mixed model (LMM) was applied to account for within-subject correlation and to examine time × subtype interactions. All model estimates are presented along with 95% confidence intervals. Statistical analyses were performed using SAS software (version 9.4; SAS Institute Inc., Cary, NC, USA).

## 3. Results

### 3.1. Tumor Characteristics

Out of a total of 116 breast cancers, 50 (43.1%) were ER-positive cancers, 45 (38.8%) were HER2-positive cancers, and 21 (18.1%) were TNBC. The mean age of patients in the ER-positive group was **47.0 years** (**range:** 28~69, median: 48), whereas those in the HER2-positive group and the TNBC group had mean ages of **52.1 years** (**range:** 28~69, median: 48) and **48.5 years** (**range:** 28~69, median: 48), respectively. Table 1 presents the clinicopathological features of tumors stratified by molecular subtype. When statistically significant differences were observed among the groups, post hoc pairwise comparisons were performed using Bonferroni correction. Menopausal status differed significantly among the three groups, with a higher proportion of premenopausal women in patients with ER-positive cancer compared to those with HER2-positive tumors. (*p* ≤ 0.001). Histopathological analysis showed that invasive ductal carcinoma was present in 92.2% (107/116) of the patients, invasive lobular carcinoma in 6.8% (8/116) and there was one case of squamous cell carcinoma. The yT stage differed significantly among all tumor molecular subtypes. The N stage differed significantly among the groups, with patients with HER2-positive tumors tending to have lower stage compared to those with ER-positive tumors. Out of a total of 116 patients, 31 achieved pathologic complete remission (pCR), including 1 with ER-positive cancer, 23 with HER2-positive cancer, and 7 with TNBC.

### 3.2. Background Parenchymal Enhancement Categories (BPEC)

BPE patterns on MRI were assessed by two radiologists in consensus. Baseline BPE categories were significantly lower in postmenopausal women than in premenopausal women (*p* ≤ 0.001) (Table 1). In ER-positive cancer, the distribution of BPEC 1/2/3/4 was 26/16/28/30% before and 68/28/4/0% after NAC (Table 2). In HER2-positive cancer, the distribution of BPEC 1/2/3/4 was 37.8/26.7/22.2/13.3% before and 73.3/20.0/6.7/0 after NAC. In TNBC, the distribution of BPEC 1/2/3/4 was 47.6/14.3/23.8/14.3% before and 76.2/14.3/9.5/0 after NAC. (Table 3).

**Figure 2:**
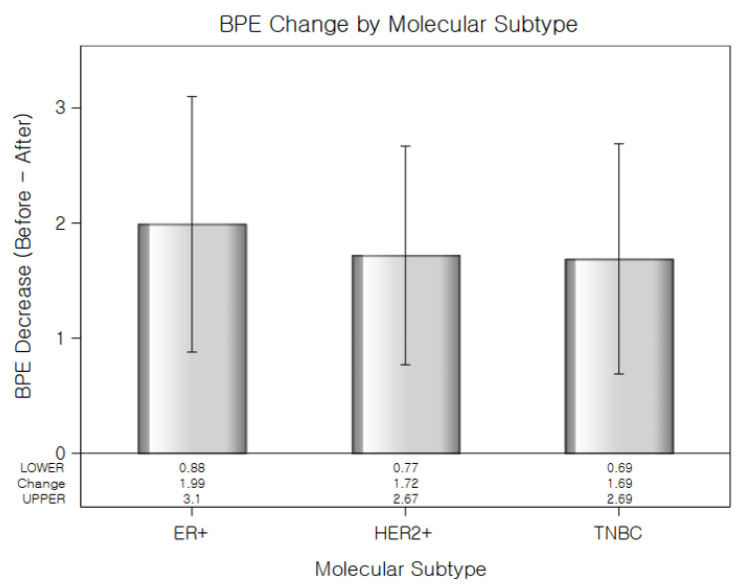
Reduction in background parenchymal enhancement levels (Before–After) following NAC for each molecular subtype. TNBC showed the largest decrease in BPE. Error Bars indicate standard error.

**Figure 3:**
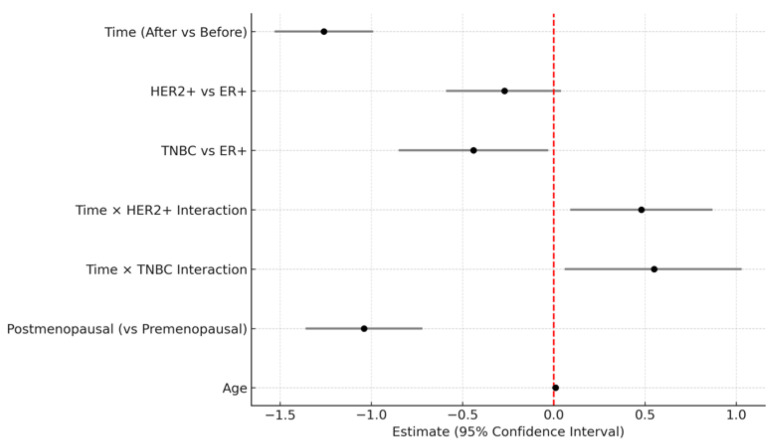
Forest plot of fixed-effect estimates and 95% confidence intervals from the linear mixed model (LMM) evaluating background parenchymal enhancement changes. The vertical red dashed line indicates no effect (estimate = 0).

**Figure 4:**
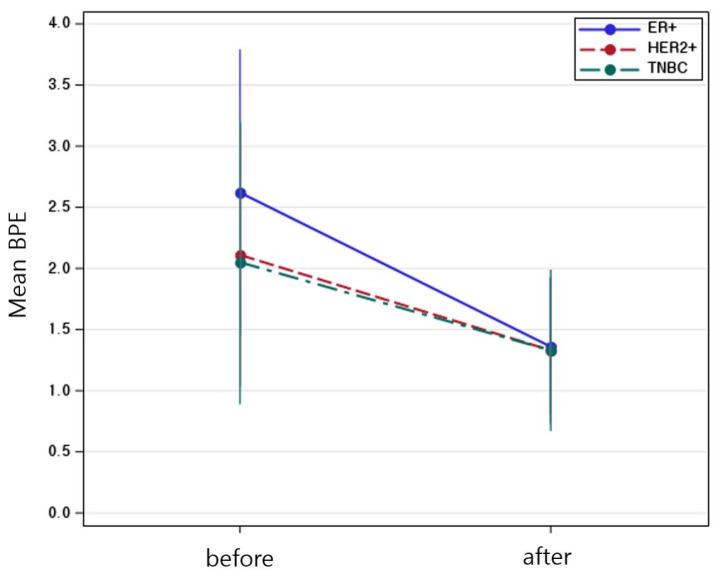
Mean background parenchymal enhancement values before and after neoadjuvant chemotherapy, stratified by molecular subtype. Error Bars indicate standard error.

**Figure 5:**
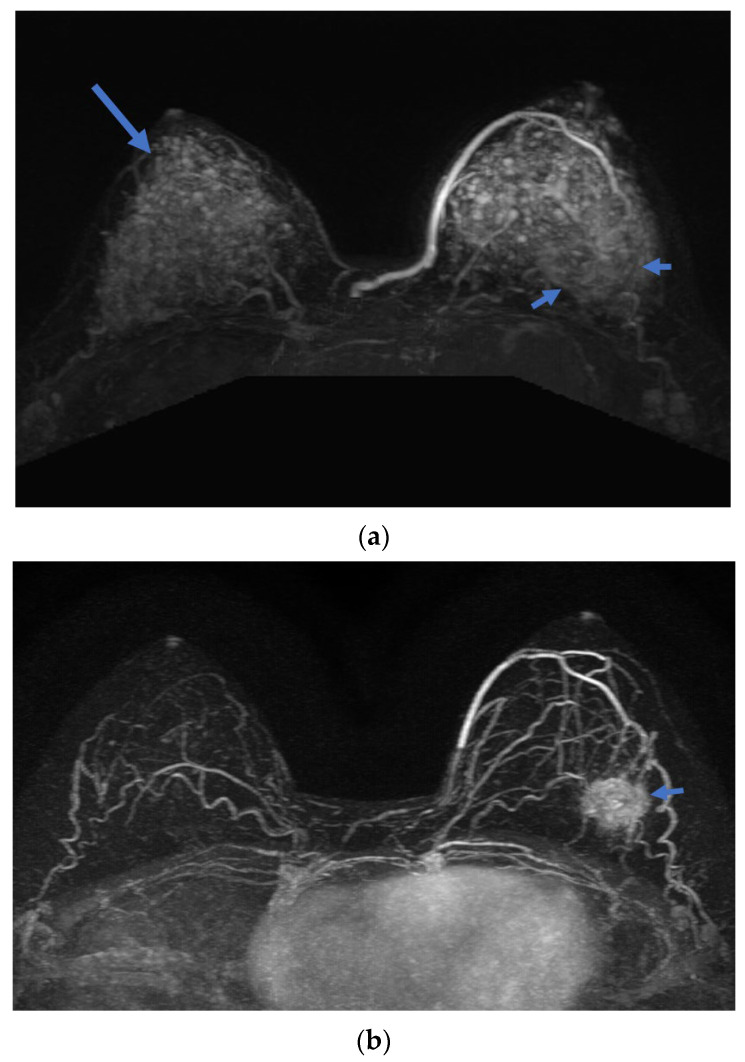
A 32-year-old woman with TNBC at left breast left 4 O`clock direction. Axial Maximal intensity projection (MIP) images with subtraction of breast MR show (**a**) baseline and (**b**) post-NAC scans. The BPE grade decreased from 4 (long arrow) at baseline to 1 after NAC, demonstrating marked reduction in BPE along with a decrease in tumor size (short arrow).

Table 4 showed a significant main effect of time (Estimate = −1.26, *p* < 0.001), indicating that BPE significantly decreased after NAC. The interaction between time and molecular subtype was also statistically significant. Compared to the ER-positive cancer, the reduction in BPE over time was significantly greater in the HER2-positive cancer group (Estimate = 0.48, *p* = 0.0168) and TNBC (Estimate = 0.55, *p* = 0.0321), suggesting that the extent of BPE decrease varied by subtype (Figure 2). No significant difference in baseline BPE was observed across subtypes. Postmenopausal status was independently associated with lower BPE values (Estimate = −1.04, *p* < 0.001), while age was not a significant factor. All molecular subtypes showed a significant reduction in BPE after NAC, with the TNBC subtype exhibiting the greatest change (Figure 3). The interaction plot demonstrated a consistent downward trend in BPE post-NAC, with TNBC showing the steepest decline (Figure 4). When comparing the change scores of BPE before and after NAC, the TNBC subtype showed the largest mean reduction (2.0), followed by HER2+ (1.1) and ER+ (0.7) subtypes (Figure 5). The reduction was statistically significant across all subtypes (*p* < 0.05).

## 4. Discussion

In this study, there is no significant difference in baseline BPE levels before NAC according to the tumor subtype. Although previous large retrospective cohort and case–control studies [6,7] have shown a correlation between BPE and breast cancer risk, our study observed no significant variation in baseline BPE across tumor subtypes prior to chemotherapy. This finding is consistent with the results reported by Benvennati and Kim. Such differences from large-scale epidemiologic risk studies may reflect variations in sample size, study design, and population characteristics, particularly given that our cohort consisted of patients already diagnosed with breast cancer.

Several retrospective cohort studies have evaluated baseline BPE and its changes during NAC in relation to treatment response, collectively demonstrating that a reduction in BPE from pre- to post-treatment MRI is significantly associated with achieving a pathologic complete response (pCR). This association is observable as early as after the second cycle of NAC and is also noted in patients receiving HER2-targeted NAC [18,19]. Previous studies have reported that ER-positive breast cancers generally demonstrate lower rates of pathologic complete response (pCR) to neoadjuvant chemotherapy compared with HER2-positive or triple-negative breast cancers [20,21]. In a large cohort of 13,939 patients, Haque et al. demonstrated an overall pCR rate of about 19%, the lowest in luminal A (0.3%) and the highest in HER2-positive cancer (38.75%) [20]. Houvenaeghel et al. reported a pCR rate of 31.7% (365/1150) with significantly different rates according to tumor subtypes, the lowest in luminal A (7.1%) and the highest in the TNBC (38.1%) [21]. Previously Chen et al. evaluated the association between BPE and pathologic response to NAC in 46 patients with unilateral invasive breast cancer, and there was significantly decreased BPE at follow up MR after neoadjuvant chemotherapy in ER-negative pCR group [17]. In this study, compared to the ER-positive cancer, the reduction in BPE over time was significantly greater in the HER2-positive cancer group (Estimate = 0.48, *p* = 0.0168) and TNBC (Estimate = 0.55, *p* = 0.0321), suggesting that the extent of BPE decrease varied by subtype. Furthermore, the TNBC subtype demonstrated the greatest mean reduction in BPE following NAC (2.0), whereas the HER2+ and ER+ subtypes exhibited comparatively smaller decreases of 1.1 and 0.7, respectively. In patients with TNBC and HER2-positive cancer, achieving a pCR after NAC is strongly associated with improved event-free survival and overall survival [22]. Given that pCR represents a key prognostic indicator particularly in patients with TNBC, early tumor response to neoadjuvant chemotherapy may carry important implications for subsequent therapeutic decision-making. Accordingly, although further validation is warranted, our findings may contribute meaningful preliminary evidence supporting the clinical value of early treatment-related BPE changes within this context. Although the pCR rate in our cohort was relatively low and the limited sample size made it difficult to analyze whether BPE changes differed significantly across molecular subtypes among patients who achieved pCR, our study nevertheless represents a meaningful exploratory effort. Given that early treatment response during neoadjuvant therapy has been shown to correlate with the likelihood of achieving pCR, our findings suggest that early BPE reduction may hold potential as a predictive imaging marker in this context. Further studies involving larger patient cohorts and quantitatively assessed changes in BPE are warranted to validate these findings.

Our study has several limitations. First, Breast MRI examinations could not be consistently scheduled between days 7 and 13 of the menstrual cycle, as timely imaging was prioritized to avoid any delays in treatment. Additionally, since this study was retrospective, the exact menstrual cycle phase at the time of MRI acquisition could not be accurately documented for each patient. Second, different chemotherapy regimens were administered according to the hormonal status of the tumor. Since BPE is influenced by hormonal factors, variations in treatment protocols may have contributed to the observed differences in BPE reduction. Third, this was a single-center retrospective study with a relatively small sample size, which may limit the generalizability of our findings. Fourth, BPE reduction was qualitatively assessed by two experienced radiologists, and no quantitative measurement was performed.

In conclusion, our study demonstrated that, in patients undergoing NAC, the degree of BPE reduction on pre- and post-treatment breast MRI significantly differed according to molecular subtypes. These findings indicate that changes in BPE may reflect the tumor’s biological features, such as molecular subtype, and suggest that understanding BPE dynamics could play a role in guiding personalized treatment planning for breast cancer patients. Further prospective and multicenter studies are warranted to validate these observations and explore their potential clinical applications.

## Figures and Tables

**Table 1:** Clinicopathological features of breast cancer according to molecular subtype.

	ER-Positive (*n* = 50)	HER2-Positive (*n* = 45)	TNBC (*n* = 21)	*p* Value
Menopausal status				0.040
Pre-menopausal	33 (66)	18 (40)	11 (52.4)	
Post-menopausal	17 (34)	27 (60)	10 (47.6)	
Operation type				0.990
Partial mastectomy	35 (70)	32 (71.1)	15 (71.4)	
Total mastectomy	15 (30)	13 (28.9)	6 (28.6)	
Pathology				0.086
Invasive ductal carcinoma	44 (88)	43 (95.6)	20 (95.2)	
Invasive lobular carcinoma	6 (12)	2 (4.4)	0 (0)	
Squamous cell carcinoma	0	0	1 (4.8)	
yT stage				<0.001
T0	1 (2)	23 (51.1)	7 (33.3)	
Tis	4 (8)	5 (11.1)	1 (4.8)	
T1	7 (14)	13 (28.9)	3 (14.3)	
T2	30 (60)	2 (4.4)	5 (23.8)	
T3	7 (14)	2 (4.4)	4 (19)	
T4	1 (2)	0 (0)	1 (4.8)	
yN stage				0.001
N0	20 (40)	36 (80)	14 (66.7)	
N1	22 (44)	7 (15.6)	5 (23.8)	
N2	7 (14)	1 (2.2)	1 (4.8)	
N3	1 (2)	1 (2.2)	1 (4.8)	

**Table 2:** The distribution of background parenchymal enhancement categories (BPEC) in premenopausal and post-menopausal patients on preoperative breast MR.

	BPEC				*p* Value
	1	2	3	4	<0.001
Pre-menopausal	6 (9.7)	9 (14.5)	23 (37.1)	24 (38.7)	
Post-menopausal	34 (63.0)	14 (25.9)	6 (11.1)	0 (0.0)	

**Table 3:** Background parenchymal enhancement category (BPEC) on breast MR according to the molecular subtype.

	ER-Positive (*n* = 50)	HER2-Positive (*n* = 45)	TNBC (*n* = 21)	*p* Value
BPEC on preoperative MR				0.237
1	13 (26)	17 (37.8)	10 (47.6)	
2	8 (16)	12 (26.7)	3 (14.3)	
3	14 (28)	10 (22.2)	5 (23.8)	
4	15 (30)	6 (13.3)	3 (14.3)	
BPEC on follow up MR				0.650
1	34 (68)	33 (73.3)	16 (76.2)	
2	14 (28)	9 (20.0)	3 (14.3)	
3	2 (4)	3 (6.7)	2 (9.5)	
4	0 (0)	0 (0)	0 (0)	

**Table 4:** Linear mixed-model analysis of Background Parenchymal Enhancement (BPE) changes after Neoadjuvant Chemotherapy (NAC).

Effect	Estimate	95% CI	*p*-Value
Time (After vs. Before)	−1.26	(−1.53, −0.99)	<0.001
Subtype (HER2+ vs. ER+)	−0.27	(−0.59, 0.04)	0.0983
Subtype (TNBC vs. ER+)	−0.44	(−0.85, −0.03)	0.0335
Time × HER2+ Interaction	0.48	(0.09, 0.87)	0.0168
Time × TNBC Interaction	0.55	(0.06, 1.03)	0.0321
Age	0.01	(−0.01, 0.02)	0.4208
Menopausal state (Post vs. Pre)	−1.04	(−1.36, −0.72)	<0.001

ER+ = estrogen receptor positive, HER2+ = human epidermal growth, TNBC = reference group; CI = confidence interval.

## Data Availability

The data presented in this study are available on request from the corresponding author.

## Supplementary Materials

The following supporting information can be downloaded at: https://www.mdpi.com/article/10.3390/diagnostics15222826/s1, Table S1: Interobserver agreement for background parenchymal enhancement grading before and after neoadjuvant chemotherapy (NAC).
