# Supplementary material for: Comparison of Contralateral Breast Background Parenchymal Enhancement on MRI Before and After Neoadjuvant Chemotherapy According to Molecular Subtypes in Unilateral Breast Cancer
Source: Diagnostics (Basel). 2025 Nov 7;15(22):2826. doi: 10.3390/diagnostics15222826 (PMC12651529; doi:10.3390/diagnostics15222826)
Supplement: Supplementary file 1 [file diagnostics-15-02826-s001.zip › diagnostics-3919808-supplementary.pdf]

Supplemental Materials

Table S1. Interobserver agreement for background parenchymal enhancement grading before and after neoadjuvant chemotherapy (NAC).

Time Point	Simple κ (95% CI)	Weighted κ (95% CI)	Interpretation
Before NAC	0.578 (0.465–0.691)	0.739 (0.660–0.818)	Substantial
After NAC	0.543 (0.358–0.728)	0.564 (0.390–0.738)	Moderate

CI = confidence interval.
